# Are cesarean deliveries equitable in India: assessment using benefit incidence analysis

**DOI:** 10.1186/s12913-022-07984-6

**Published:** 2022-05-18

**Authors:** Rajeev Ranjan Singh, Suyash Mishra, Sanjay K. Mohanty

**Affiliations:** 1 International Institute for Population Sciences, Mumbai, India; 2 Department of Population and Development, International Institute for Population Sciences, Mumbai, India

**Keywords:** Cesarean delivery, Equity, Inequality, Benefit Incidence Analysis, India

## Abstract

**Background:**

In the last two decades, cesarean section (CS) deliveries in India have increased by six-fold and created economic hardship for families and households. Although several schemes and policies under the National Health Mission (NHM) have reduced the inequality in the use of maternal care services in India, the distributive effect of public health subsidies on CS deliveries remains unclear. In this context, this paper examines the usage patterns of CS delivery and estimates the share of public health subsidies on CS deliveries among mothers by different background characteristics in India.

**Data:**

Data from the fourth round of the National Family Health Survey (NFHS-4) was used for the study. Out-of-pocket (OOP) payment for CS delivery was used as a dependent variable and was analyzed by level of care that is, primary (PHC, UHC, other) and secondary (government/municipal, rural hospital). Descriptive statistics, binary logistic regression, benefit incidence analysis, concentration curve and concentration index were used for the analysis.

**Results:**

A strong economic gradient was observed in the utilization of CS delivery from public health facilities. Among mothers using any public health facility, 23% from the richest quintile did not pay for CS delivery compared to 13% from the poorest quintile. The use of the public subsidy among mothers using any type of public health facility for CS delivery was pro-rich in nature; 9% in the poorest quintile, 16.1% in the poorer, 24.5% in the middle, 27.5% among richer and 23% in the richest quintile. The pattern of utilization and distribution of public subsidy was similar across the primary and secondary health facilities but the magnitude varied. The findings from the benefit-incidence analysis are supported by those obtained from the inequality analysis. The concentration index of CS was 0.124 for public health centers and 0.291 for private health centers. The extent of inequality in the use of CS delivery in public health centers was highest in the state of Mizoram (0.436), followed by Assam (0.336), and the lowest in Tamil Nadu (0.060), followed by Kerala (0.066).

**Conclusion:**

The utilization of CS services from public health centers in India is pro-rich. Periodically monitoring and evaluating of the cash incentive schemes for CS delivery and generating awareness among the poor would increase the use of CS delivery services in public health centers and reduce the inequality in CS delivery in India.

**Supplementary Information:**

The online version contains supplementary material available at 10.1186/s12913-022-07984-6.

## Background

The increasing prevalence of cesarean delivery, its associated costs, and the growing health inequalities are public health challenge globally [1]. While cesarean section (CS) is a globally accepted life-saving surgical technique to deal with pregnancy complications and reduce maternal and neonatal mortality and morbidity, an excessive use of cesarean deliveries is becoming a new normal in many developing countries. Based on data from 169 countries, recent global estimates suggest prevalence of CS deliveries of 21.1% which is in excess of the WHO limit of 10–15% of all births [2]. Reasons for increasing cesarean deliveries are many: multiple pregnancies, pregnancy complications, dystocia, foetal distress, repeated cesarean birth, increased maternal body mass index (BMI), rising mother’s age, fear of vaginal delivery, increase in institutional delivery misuse of cesarean sections in private health centers and avoidable cesarean sections [3–6]. Goal 3.7 of the Sustainable Development Goals (SDGs) aims at achieving universal access to maternal and reproductive health services, whereas Goal 3.8 aims to achieve universal health coverage, financial risk protection and access to quality health services by 2030 [7, 8]. The progress in attaining SDGs is contingent on the availability of affordable and quality maternal care services including CS delivery.

**Fig. 1:**
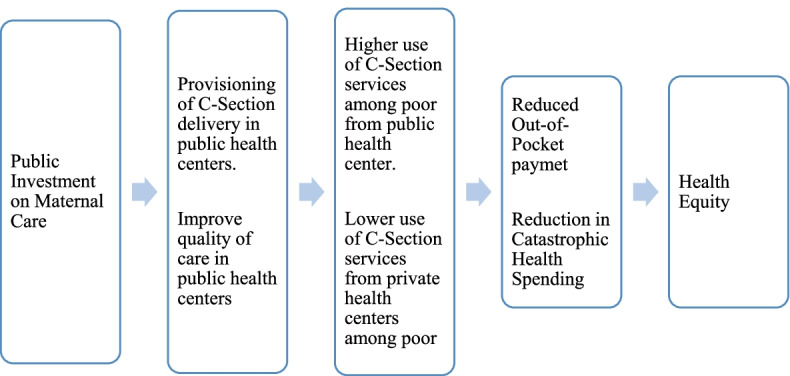
Conceptual framework on effect of public investment on maternal care

Among other factors, cesarean births are associated with higher out-of-pocket (OOP) payments, catastrophic health expenses (CHS), and increased financial burden on households. While CS deliveries are expensive, some are unavoidable. In a welfare state, public investment in basic health care such as maternal care is likely to make available life-saving services such as CS delivery and improve the quality of maternal care in public health centers. These, in turn, would increase the use of CS delivery from public health centers and lower their use from private health centers. A larger use of CS delivery care from public health centers can reduce out-of-pocket (OOP) expenditure and financial catastrophe for households and help in achieving health equity (**Fig. ****1**).

Various approaches are used to examine the equity of public health services. These include benefit incidence analyses, concentration curves and indices, and the behavioral approach. Studies examining the distribution of public subsidies on health care services using the benefit incidences analysis are limited. In Kenya, public subsidies on primary health centers were found to be benefiting the poorest section of the population; however, at the hospital level, the benefits were mostly pro-rich [9]. Examining the incidence of public spending on health care in 11 Asian countries, it was found that the benefits received for health care services were evenly distributed across wealth group in Sri Lanka and Thailand, whereas they were pro-poor in Hong Kong and Malaysia. In India, Bangladesh and Indonesia, more than one third of the benefits associated with using public health services were availed by the richest segment of the population [10]. Studies from Ghana, Malwi and other low and middle income counties (LMICs) [11–13] found that the policy to exempt the user fee resulted in an increased utilization of facility-based deliveries, including CS deliveries, and a reduction in socio-economic inequality. In India, the share of public subsidies on delivery care services is skewed towards the more affluent sections of the population [14].

The National Health Mission (NHM), one of the most extensive health programs globally, has been successful in reducing maternal and child mortality in the country as well as increasing institutional deliveries [15]. Janani Suraksha Yojana (JSY), Janani Shishu Suraksha Karyakaram (JSSK) and Pradhan Mantri Matru Vandana Yojana (PMMVY) are centrally sponsored schemes under NHM that concern themselves with institutional delivery including cesarean section. While JSY and PMMVY provide monetary assistance in the form of conditional cash transfer for institutional delivery to poor mothers, JSSK ensures free and cashless services to pregnant mothers, including for cesarean sections. The existing literature provide inconclusive evidence regarding the effectiveness of programs. While some studies argue that the programs have been effective in reduction of socio-economic disparities in institutional delivery, along with cesarean sections [16–18] others argue conversely [19–21].

Evidence suggests that institutional deliveries in India increased from 39% in 2005–06 to 89% in 2019–21 [22, 23], CS deliveries too increased significantly from 8.5% to 21.5% during this period.  However, the OOP expenditure on CS delivery is high and varies significantly across the states of India, by socio-economic gradients, type of healthcare facility (public/private) and other factors. A recent study suggests that the expenditure on CS delivery in a private health facility is three times higher than in a public health facility [24]. A rising number of cesarean births, higher use of private health centers and higher cost of cesarean delivery are some of the common features of low and middle-income countries (LMICs). In India too, cesarean deliveries are increasing at an accelerated rate, some of which are induced by providers in private health centers and are totally unwarranted. In public health centers, cesarean delivery services available only in district hospitals and primary health centers and not in all public health facilities. Though national guidelines on the practice of cesarean delivery exist, these are rarely practiced by the health care providers specially in the private health facilities.

While India has significantly improved its national indicators over time, inequality in health care service remains large [25]. Public health facilities in India account for only one-third of the CS deliveries conducted. and even in those facilities, the existing literature reveals the use of cesarean delivery is higher among the richer sections of the population [26, 27]. Despite the increase in CS deliveries in public health facilities over time, little is known about who the beneficiaries of the services are and whether the subsidy benefits are pro-poor or pro-rich in nature[28–32]. None of the studies has quantified the extent of public subsidy on CS delivery in India either. The present study estimates the benefit-incidence of public subsidies on CS delivery in India.

## Methods

### Data

Micro data (individual records) from the fourth round of the National Family Health Survey (NFHS-4) conducted in 2015–16 was used for the analysis. NFHS-4 is a cross-sectional nationally representative survey conducted under the aegis of the Ministry of Health and Family Welfare, Government of India to provide reliable estimates of maternal and child health indicators, nutrition, contraception etc. at the state and district level. The survey uses multilevel stratified sampling, taking the 2011 Census as the sampling frame for selecting the Primary Sampling Units (PSUs), which are villages in the case of rural areas and Census Enumeration Blocks (CEBs) in the case of urban areas. The survey collected information from 601,509 households, 699,686 unmarried women in the age group 15–49 year and 112,122 men in the age group 15–54 years across India. Uniquely, the survey pioneered the practice of collecting information on out-of-pocket (OOP) payments for the last birth delivered in a health facility. Data on OOP payment was edited for probable errors before tabulation. The findings, methodology and the sample design of the survey are available in the national report [33].

The NFHS-4 children's file provides information on births to women during five years prior to the survey. A total of 259,627 births were reported, of which 148,645 were last births delivered in a health facility. Of these, 29,738 births were cesarean section. Although information on cesarean delivery was collected for all births during the last five years before the survey date, information on OOP payments was sought only for previous births during the same period. Last birth to a mother during the five years preceding the survey was the unit of analysis. Services received from primary health centers [PHCs], urban health centers [UHCs], urban family welfare centers [UFWCs], and urban primary Health Center [UPHC] were classified as primary care, while services received from government/municipal hospitals and rural hospitals were classified as secondary care.

### Methodology

Descriptive analysis, binary logistic regression, benefit incidence analysis (BIA), concentration index (CI), and concentration curve (CC) were used in the analysis. The analysis was carried out in three stages; a) In the first stage, we identified the predictors of cesarean delivery in public health centers b) In the second stage, we performed the benefit-incidence analysis c) In the third stage, we estimated the concentration indices and concentration curves.

### Variables

Use of cesarean delivery services at public health centers was the dependent variables. The independent variables were wealth quintile, place of residence (rural/urban), low and high performing states depending on the extent of institutional delivery; mother's age (15–24 years, 24–34 years and 35 and over); mother's level of education (less than five years, more than five years); number of ANC visits (less than 4 visits, 4 or more visits) and social group (Scheduled Caste [SC], Scheduled Tribe [ST], Other Backward Class [OBC], and others. The SC, ST and OBC groups are considered socially disadvantaged section of the population in India and they are given reservations in education and employment and many other benefits by the national, state and local governments. We used OOP expenditure on cesarean births for estimation in BIA. Data on OOP in NFHS-4 was collected at current prices over a period of seven years. A direct comparison of prices over a period of five years would not have provide true estimates; so, data on OOP payments was adjusted using the Consumer Price Index (CPI). The CPI for rural and urban areas and for each state from January 2011 to December 2016, published by the Government of India on a monthly basis, was used to adjust the OOP data [34]. We made OOP payments estimates at 2016 price (December).

### Binary logistic regression

We identified the significant predictors of cesarean delivery in public health centers based on logistic regression analysis. The dependent variable was coded as 1 if a mother has had a CS delivery and as 0 otherwise. The general form of the regression model is as follows:

$$logit (\pi_i) = a + \beta_1 (place of residence_i) + \beta_2 (age_i) + \beta_3 (education level_i) + \beta_4 (social group_i) + \beta_5 (state type_i) + \beta_6 (household size_i) + \beta_7 (antental care visit_i) + \beta_8 (wealth quintile_i) + \beta_9 (pregnacy complication_i) + e_i. (1)$$ 

where ${\uppi }_{i}$ is the probability of having a cesarean delivery in a public health center, α is the intercept and β ‘s are the slope parameter.

### Benefit incidence analysis

We used the benefit-incidence analysis (BIA) to estimate the extent of inequality in the distribution of public subsidy on cesarean delivery across socio-economic groups and by type of public health centers., Benefit incidence analysis is a tool to measure whether government-funded health subsidies benefit public health equitably. The underlying assumption of the BIA is that public spending and services provided should benefit those belonging to the low socioeconomic strata. With a growing emphasis on the need for pro-poor health funding, BIA has become a well-established method to investigate the benefits of public health subsidies. One of the difficulties in estimating BIA relates to obtaining the actual cost of service for cesarean delivery. In the absence of an actual cost of service, studies have used the mean, median or modal values of OOP in private healthcare facilities as a proxy for the cost of service [14, 30, 35–39]. However, since the data is heterogeneous and includes a large number of null values, using the mean and modal values is not appropriate. So we preferred using the median value of OOP in private health centers as the cost of service in a public health facility.

The steps used in estimating BIA for cesarean delivery are given below:

1. Computation of wealth quintile (individuals ranked by wealth) to measure the socio-economic status.
2. Estimation of utilization rate for CS delivery in public health centers by wealth quintile.
3. Calculation of net subsidy (by subtracting the median OOP in public health facilities from the median OOP in private health centers).
4. Multiplication of the net subsidy with the utilization rate for each wealth quintile to compute individual subsidy.
5. Calculation of benefit incidence by taking percentage share of each quintile to the total subsidy.

The benefit incidence analysis was estimated for a group ‘*g’* utilising CS delivery service ‘*s’* in a public health center. Cost of service in public health facility was substituted with OOP in private health facility.

Mathematically, the Benefit Incidence is defined as follows:

$${\mu }_{g} = \sum {\alpha }_{sg}\frac{{\beta }_{s}}{{\alpha }_{s}}\bullet \bullet =\sum {\gamma }_{sg}{\beta }_{s}$$

where.

${\mu }_{g}=$ Benefit of public subsidy utilized by group g.

${\alpha }_{sg}=$ Utilization of CS delivery care (s) by group g.

${\alpha }_{s}=$ Utilization of delivery care (s) by all groups.

${\beta }_{s}=$ Net expenditure on CS delivery (s) by government.

${\gamma }_{sg}=$ Group (g) share of utilization of CS delivery care (s).

### Concentration curve and concentration index

Public health researchers have been increasingly using concentration curve (CC) and concentration index (CI) to understand economic inequality in relation to the health outcome of interest [40–43]. The CC refers to the progressive proportion of the population based on wealth versus the progressive population using CS delivery care services in health facilities (public or private). The CC plots below the line of equality show a pro-rich use of services. In contrast, CC plot above the line of inequality shows a pro-poor use of services, the coincidence of the CC plot with the line of equality shows an equitable use of services among the wealth quintiles. CI are derived from CC and its value ranges from -1 to + 1, A zero value represents uniform distribution [44].

## Results

**Fig. 2:**
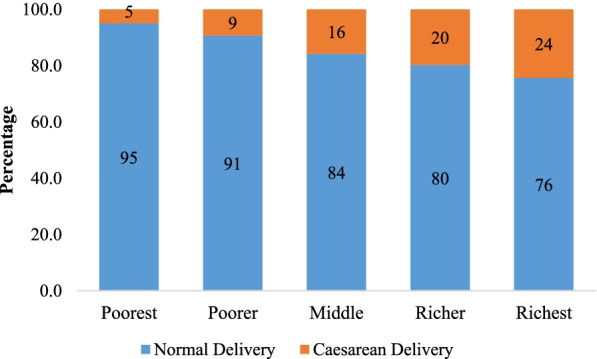
Percent distribution of normal and cesarean delivery in public health facility by wealth quintile in India 2015–16

**Figure ****2** presents the percent distribution of cesarean and normal deliveries in public health facilities by wealth quintile in India. The utilization of cesarean delivery care in public health centers has a strong economic gradient; lower among the poorest and poorer wealth quintile and higher among the richest and richer wealth quintile for instance, in the poorest wealth quintile, only 5% mothers used cesarean delivery compared to 24% in the richest wealth quintile.

**Table****1** shows the socio-demographic profile of the mothers who used CS delivery by type of health facility in India. Out of the total mothers who used CS delivery care in public health facilities, 42% belonged to urban area while 58% belonged to rural areas. About 17% of the mothers had less than five years of schooling while 83% had more than five years of education. About 30% of the mothers resided in low performing states while 70% resided in high performing states. With regard to social group, 31% of the mothers belonged to scheduled caste/tribe, 39% belonged to other backward classes and 30% belonged to other social group. Among mothers utilizing services at a public health facility, 89% did so at a government/municipal facility; or a rural hospital, while 11% relied on PHCs, UHCs and others. About 72% of the mothers made 4 or more ANC visits while 28% less than 4 ANC visits. About 57% of mothers had some pregnancy complication. The pattern was similar with varying magnitude among mothers using private health facilities for CS delivery. For instance, about 88% had more than five years of education while 12% had less than five years of education.

**Table 1:** Sample profile of the study population used cesarean delivery in public and private health centers based on NFHS-4, India, 2015–16

Variables	Public		Private	
	**Percentage**	**N**	**Percentage**	**N**
**Place of Residence**				
Urban	42.5	5,375	49.5	8,458
Rural	57.5	7,271	50.5	8,634
**Level of Education**				
Less than 5 years	16.7	2,115	11.7	1,998
5 years and above	83.3	10,531	88.3	15,094
**State type**				
Low Performing states	30.4	3,845	30.6	5,228
High performing state	69.6	8,801	69.4	11,864
**Social group**				
Schedule caste/Schedule tribe	31.3	3,961	18.5	3,167
Other backward class	38.6	4,882	46.4	7,932
others	30.1	3,803	35.1	5,993
**Level of care at public health centers**				
Government/municipal, Rural Hospital	88.8	11,229	NA	NA
PHC, UHC, others	11.2	1,417	NA	NA
**Mother’s Age**				
15–24	36.5	4,611	31.1	5,323
25–34	56.7	7,174	59.9	10,236
35 +	6.8	8,61	0.9	1,534
**Number of ANC visits**				
Less than 4	27.9	3,534	25.3	4,316
4 and above	72.1	9,112	74.7	12,776
**Pregnancy Complication**				
No	42.7	5,691	47.1	7,991
Yes	57.3	6,955	52.9	9,101

**Table ****2** presents the results of the binary logistic regression for cesarean delivery in public health facilities in India. Place of residence, mother’s age and educational level, social group, number of ANC visits, economic status and pregnancy complication were found to be significant predictors of CS delivery in public health facilities of India. The odds of availing CS delivery care in a public health facility were 2.7 times (AOR: 2.74; 95% CI: 2.51–2.99) higher among mothers belonging to the richest wealth quintile compared to those from the poorest wealth quintile. The odds of CS delivery among mothers belonging to high performing states were 1.4 times (AOR: 1.43; 95% CI: 1.37–1.49) higher compared to those belonging to low performing states. With regard to place of residence, the odds of CS delivery were 1.3 times (AOR: 1.27; 95% CI: 1.21–1.33) higher in urban areas compared to rural area. Mothers with five or more years of education were 1.5 (AOR: 1.47; 95% CI: 1.39–1.55) times more likely to have CS delivery compared to those having less than 5 years of education. In contrast, mothers belonging to the SC/ST social group were 0.61 (AOR: 0.61; 95% CI: 0.58–0.64) times less likely to experience CS delivery compared to those belonging to the “other” social group.

**Table 2:** Adjusted odds ratio of cesarean delivery in public health facility in India, 2015–16

Variables	AOR	95% Confidence Interval
**Wealth Quintile**		
Poorest ®		
Poorer	1.44***	[1.33–1.55]
Middle	2.09***	[1.94–2.26]
Richer	2.51***	[2.31–2.72]
Richest	2.74***	[2.51–2.99]
**Place of Residence**		
Rural ®		
Urban	1.27***	[1.21–1.33]
**State Type**		
Low Performing States ®		
High Performing States	1.43***	[1.37–1.49]
**Mother's Education Level**		
Less than 5 years ®		
5 years and above	1.47***	[1.39–1.55]
**Social Group**		
Others ®		
ST/SC	0.61***	[0.58–0.64]
OBC	0.64***	[0.61–0.67]
**Mother's Age**		
15–24 ®		
25–34	1.13***	[1.09–1.18]
35 +	1.38***	[1.29–1.49]
**ANC Visit**		
Less than 4 ®		
ANC 4 +	1.83***	[1.75–1.91]
**Pregnancy Complication**	1.08***	[1.04–1.12]
No		
Yes		
*** p-value < 0.01, **p-value < 0.05, *p-value < *0.10*		

**Table ****3** presents the percent distribution of mothers who availed CS delivery with and without payment by wealth quintile and type of health facility in India. Overall, 11.6% mothers in India did not pay for CS delivery. The figure varied from 8.7% in the poorest quintile to 12.6% in the richer quintile. A strong economic gradient was observed among mothers who did not pay for CS delivery in both public and private health facilities however, the magnitude was higher in public health facilities compared to private health facility. For example, among mothers who availed services from a public health facility, 13.1% from the poorest quintile did not pay for the service compared to 22.9% from the richest quintile. Similarly, among mothers utilizing private health facilities, 4.2% from the poorest quintile did not pay for the service compared to 8.8% from the richer quintile. The pattern of non-payment for CS delivery remained similar with a varying magnitude when the analysis was stratified by level of care in public health facilities. For instance, among mothers who availed cesarean delivery service from PHCs, UHCs and others facilities, about 12.7% from the poorest quintile did not pay for the cesarean delivery services compared to 22.7% from the richest quintile. Similarly, among mothers utilizing services from a government/municipal facility, or a rural hospital, 16% of those from the poorest wealth quintile did not pay for the cesarean delivery compared to 25.2% of those from the richest quintile.

**Table 3:** Percent distribution of mothers who paid and did not pay for cesarean delivery by wealth quintile and type of health centers in India, 2015–16

Wealth Quintile	PHC,UHC & others*			Government/Municipal, Rural Hospital			Any Public Health Facility			Private Health Facility			Overall		
	**Paid (%)**	**Didn't Pay (%)**	**N**	**Paid (%)**	**Didn't Pay (%)**	**N**	**Paid (%)**	**Didn't Pay (%)**	**N**	**Paid (%)**	**Didn't Pay (%)**	**N**	**Paid (%)**	**Didn't Pay (%)**	**N**
**Poorest**	87.3	12.7	1002	84	16	111	86.9	13.1	1113	95.8	4.2	822	91.3	8.7	1,861
**Poorer**	85.8	14.2	1961	85.2	14.8	237	85.7	14.3	2202	95.5	4.5	1527	90.3	9.7	3,570
**Middle**	80.9	19.1	3022	84.2	15.8	378	81.4	18.7	3409	93.5	6.6	3082	87.9	12.1	6,324
**Richer**	81	19	3091	74.7	25.3	343	80.3	19.7	3436	91.2	8.8	4968	87.4	12.6	8,439
**Richest**	77.3	22.7	2345	74.9	25.2	156	77.1	22.9	2486	91.5	8.5	6692	88.3	11.7	9,544
**Total**	**81.6**	**18.4**	**11,422**	**80.5**	**19.5**	**1224**	**81.5**	**18.5**	**12,646**	**92.3**	**7.7**	**17,092**	**88.4**	**11.6**	**29,738**

**Table ****4** shows the utilization rate, out of pocket (OOP) payment and estimates of benefit incidence on CS delivery by level of care and wealth quintile in India. The utilization rate for cesarean delivery in primary health centers varied from 28% in richer wealth quintile to 9% in the poorest while in secondary health care, the utilization rate varied from 28% in richer quintile to 11% in poorest quintile. In case of any public health facility, the utilization rate varied from 27% in richer wealth quintile to 9% in the poorest quintile. Considering the median OOP for the cesarean delivery in private health facilities as a proxy for the cost of cesarean delivery, the share of public subsidy was found to be pro-rich in each type of public health facility. For instance, the share of public subsidy in any public health centers was the highest for the richer quintile (27.5%) followed by the middle quintile (24.5%) while it was lowest for the poorest quintile (9.0%). Similarly, the share of public subsidy in primary health centers was highest for the richer quintile (27.5%) followed by middle quintile (24.5%) while it was lowest for the poorest quintile (8.7%). With regard to secondary health centers, a similar pattern of share of public subsidy was observed.

**Table 4:** Utilization rate, out of pocket payment (₹), and benefit incidence on cesarean delivery by wealth quintile and level of care in India, 2015–16

Type of public health center	Wealth Quintile	Number of people utilizing public health services (1)	Utilization Rate (2)	Median OOP in Public Health services in ₹ (3)	Median cost of service in private health center in ₹ (4)	Net subsidy at public health center in ₹ (5 = 4–3)	Individual Subsidy Benefit (6 = 5*2)	Benefit Incidence (7)
**Primary:** **PHC, UHC, & others#**	**Poorest**	1061	0.093	4200	20,000	15,800	1468	8.7
	**Poorer**	1872	0.164	3900	20,000	16,100	2639	15.7
	**Middle**	2780	0.243	3050	20,000	16,950	4125	24.5
	**Richer**	3114	0.273	3000	20,000	17,000	4635	27.5
	**Richest**	2595	0.227	2500	20,000	17,500	3976	23.6
	**Total**	11,422					16,842	
**Secondary: Government/** **Municipal,** **Rural Hospital**	**Poorest**	133	0.109	1500	20,000	18,500	2010	12.0
	**Poorer**	242	0.198	3000	20,000	17,000	3361	20.0
	**Middle**	303	0.248	4000	20,000	16,000	3961	23.5
	**Richer**	336	0.275	2400	20,000	17,600	4831	28.7
	**Richest**	210	0.172	4500	20,000	15,500	2659	15.8
	**Total**	1224					16,823	
**Any Public** **Health Centers**	**Poorest**	1194	0.094	4000	20,000	16,000	1511	9.0
	**Poorer**	2114	0.167	3800	20,000	16,200	2708	16.1
	**Middle**	3083	0.244	3100	20,000	16,900	4120	24.5
	**Richer**	3450	0.273	3000	20,000	17,000	4638	27.5
	**Richest**	2805	0.222	2530	20,000	17,470	3875	23.0
	**Total**	12,646					16,852	

**Table ****5** shows the utilization rate, OOP and benefit incidence on cesarean delivery by place of residence, low/high performing states, educational attainment, and social group in India. The utilization rate of cesarean delivery in public health facilities in urban areas varied from 25.2% in the poorer wealth quintile to 15% in the richest wealth quintile and from 27.6% in the richer quintile to 9% in the poorest quintile. In urban area, the share of public subsidy was highest for the poorer quintile (25.6%) followed by the middle quintile (22.7%) and the lowest for the richest quintile (15.6%) In rural area, the share of public subsidy was highest for the richer quintile (28.3%) followed by richest quintile (27.1%) and the lowest for the poorest quintile (8.7%). The utilization rate of public health facilities in low performing states varied from 29% in the richer quintile to 8.4% in the poorest quintile while in high performing states in varied from 15.5% in the richest quintile to 23.5% in the middle quintile. The share of public subsidy in LPS was pro-rich in nature. In the case of HPS, the share of public subsidy was highest for the middle quintile (24.1%) and the lowest in the richest quintile (15.9%). The utilization rate of public health facilities among mothers with less than 5 years of education varied from 32.5% in the richest quintile to 9.7% in the poorest quintile Among mothers with education more than 5 years varied from 13.2% in the poorest quintile to 24.4% in the middle quintile. With respect to social group, the utilization rate varied from 28.7% in the richest quintile to 8.2% in the poorest quintile among mothers from scheduled castes/tribes, whereas it varied from 27.7% in the richer quintile to 9.9% in the poorest quintile among mothers from OBCs. Among mothers from other social group, the utilization rate varied from 13.9% in the poorest wealth quintile to 24.7% in the middle quintile. The share of public subsidy among respondent belonging to scheduled caste/tribe and OBCs was pro-rich in nature however, in case of mothers from other social groups, the share of the subsidy was the highest for the middle quintile (25.3%). With regard to age of mother, number of ANC visits and pregnancy complication the share of public subsidy was higher among mothers from the richer section of the population **(Additional file 1)**.

**Table 5:** Utilization rate, out of pocket payment (₹), and Benefit incidence place of residence, educational attainment, states and social group on cesarean delivery by wealth quintile in India, 2015–16

	**Wealth Quintile**	**Number of people utilizing public health services (1)**	**Utilization Rate (2)**	**Median OOP in Public Health services in ₹ (3)**	**Median cost of service in private health center in ₹ (4)**	**Net subsidy at public health center in ₹ (5 = 4–3)**	Individual Subsidy Benefit (6 = 5*2)	**Benefit Incidence (7)**
**Urban**	**Poorest**	787	0.175	3100	20,350	17,250	3023	17.2
	**Poorer**	1133	0.252	2500	20,350	17,850	4503	25.6
	**Middle**	1040	0.232	3100	20,350	17,250	3995	22.7
	**Richer**	857	0.191	3020	20,350	17,330	3307	18.8
	**Richest**	674	0.150	2100	20,350	18,250	2739	15.6
	**Total**	4491					17,567	
**Rural**	**Poorest**	730	0.090	4000	20,000	16,000	1432	8.7
	**Poorer**	1240	0.152	4000	20,000	16,000	2433	14.7
	**Middle**	1789	0.219	4000	20,000	16,000	3510	21.2
	**Richer**	2248	0.276	3000	20,000	17,000	4686	28.3
	**Richest**	2148	0.263	3000	20,000	17,000	4478	27.1
	**Total**	8155					16,539	
**LPS**	**Poorest**	505	0.084	3500	21,000	17,500	1471	8.5
	**Poorer**	797	0.133	5000	21,000	16,000	2123	12.3
	**Middle**	1225	0.204	4800	21,000	16,200	3304	19.1
	**Richer**	1742	0.290	3500	21,000	17,500	5075	29.3
	**Richest**	1738	0.289	2500	21,000	18,500	5353	30.9
	**Total**	6007					17,325	
**HPS**	**Poorest**	1204	0.181	3700	20,000	16,300	2956	17.4
	**Poorer**	1439	0.217	3030	20,000	16,970	3678	21.6
	**Middle**	1561	0.235	2600	20,000	17,400	4091	24.1
	**Richer**	1406	0.212	3100	20,000	16,900	3579	21.0
	**Richest**	1029	0.155	2530	20,000	17,470	2708	15.9
	**Total**	6639					17,012	
**Education less than 5 Years**	**Poorest**	214	0.097	3100	19,000	15,900	1538	9.9
	**Poorer**	282	0.127	4800	19,000	14,200	1809	11.6
	**Middle**	417	0.188	4000	19,000	15,000	2826	18.2
	**Richer**	580	0.262	3040	19,000	15,960	4183	26.9
	**Richest**	720	0.325	3000	19,000	16,000	5206	33.5
	**Total**	2213					15,562	
**Education more than 5 Years**	**Poorest**	1374	0.132	4000	20,000	16,000	2107	12.5
	**Poorer**	2141	0.205	3500	20,000	16,500	3386	20.1
	**Middle**	2548	0.244	3000	20,000	17,000	4152	24.6
	**Richer**	2461	0.236	3000	20,000	17,000	4010	23.8
	**Richest**	1909	0.183	2500	20,000	17,500	3202	19.0
	**Total**	10,433					16,857	
**Scheduled caste/Scheduled Tribe**	**Poorest**	346	0.082	3500	20,000	16,500	1352	8.1
	**Poorer**	597	0.141	4400	20,000	15,600	2205	13.2
	**Middle**	900	0.213	4200	20,000	15,800	3366	20.1
	**Richer**	1168	0.277	2550	20,000	17,450	4825	28.8
	**Richest**	1213	0.287	2530	20,000	17,470	5017	29.9
	**Total**	4224					16,765	
**OBC**	**Poorest**	417	0.099	3000	20,000	17,000	1689	9.7
	**Poorer**	715	0.170	3000	20,000	17,000	2895	16.6
	**Middle**	1023	0.244	2330	20,000	17,670	4306	24.6
	**Richer**	1161	0.277	2500	20,000	17,500	4840	27.7
	**Richest**	882	0.210	2100	20,000	17,900	3761	21.5
	**Total**	4198					17,491	
**Others**	**Poorest**	589	0.139	5300	20,000	14,700	2050	12.8
	**Poorer**	959	0.227	4800	20,000	15,200	3451	21.5
	**Middle**	1042	0.247	3500	20,000	16,500	4070	25.3
	**Richer**	924	0.219	3850	20,000	16,150	3533	22.0
	**Richest**	710	0.168	2300	20,000	17,700	2975	18.5
	**Total**	4224					16,079	

### Concentration curve and concentration indices of cesarean delivery

**Fig. 3:**
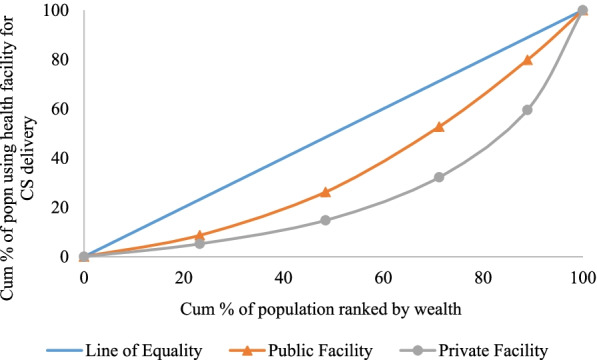
Concentration curve for mothers availing cesarean delivery in public and private health facility in India, 2015–16

**Figure ****3** presents the concentration curve (CC) for mothers who had a CS delivery by type of health center in India. The concentration curves for CS delivery in public and private health centers were below the line of equality indicating a pro-rich concentration of CS delivery. The extent of inequality was relatively higher in private health facilities compared to public health facilities.

**Table ****6** presents the concentration indices for cesarean delivery by place of residence, low and high performing states, social group, level of education, number of ANC visits and pregnancy complication in India. The CI value was positive for both public (CI: 0.124) and private health centers (CI: 0.291) suggesting a pro-rich inequality in the utilization of CS delivery services. The magnitude of the inequality was much higher in private health facilities compared to public health facilities. For each of the selected covariates, the inequality was pro-rich in nature in both public and private health facility. Among mothers who had used a public health facilities for CS delivery, the CI value was higher for those residing in rural area (CI: 0.124) compared to those who resided in urban area (CI: 0.074). The pattern remained similar for private health facilities. With regard to state type, the CI value was higher in LPS compared to HPS. For instance, the CI value for mothers who reside in LPS and availing CS delivery in public health facility was 0.194 while the CI value was 0.050 among those residing in high HPS. The inequality in using CS delivery from both public and private health facilities was higher among mothers belonging to marginalized social groups. For instance, among mothers who used a public health facility, the CI value for CS delivery was the highest among those belonging to the SC/ST (CI: 0.176) social group followed by those belonging to the OBC group (CI: 0.112) and those belonging to the “other” social group (CI: 0.096). With regard to education level, the CI value was higher for mothers having education of less than 5 years compared to those having education of 5 years and above in both public and private health centers. A higher value of CI was observed for mothers with any pregnancy complication compared to those with no complication across all types of public and private health facility.

**Table 6:** Concentration index for cesarean section delivery by selected covariates in India, 2015–16

Variable	Place of Delivery			
	**Public**	**95% CI**	**Private**	**95% CI**
**Place of Residence**				
Rural	0.122	[0.110–0.134]	0.270	[0.253–0.287]
Urban	0.074	[0.056–0.092]	0.138	[0.114–0.162]
**State Type**				
Low performing states	0.194	[0.180–0.208]	0.354	[0.332–0.376]
High performing states	0.050	[0.036–0.064]	0.129	[0.109–0.149]
**Social Group**				
SC/ST	0.176	[0.154–0.198]	0.323	[0.290–0.356]
OBC	0.112	[0.098–0.126]	0.291	[0.269–0.313]
Other	0.096	[0.078–0.114]	0.210	[0.184–0.236]
**Education**				
Less than 5 years	0.124	[0.095–0.153]	0.295	[0.252–0.338]
5 years and more	0.068	[0.056–0.080]	0.207	[0.189–0.225]
**Number of ANC visit**				
Less than 4	0.178	[0.160–0.196]	0.335	[0.301–0.369]
4 and more	0.056	[0.044–0.068]	0.193	[0.175–0.211]
**Pregnancy Complication**				
No	0.150	[0.134–0.166]	0.339	[0.315–0.363]
Yes	0.098	[0.084–0.112]	0.254	[0.232–0.276]
**Overall**	**0.124**	**[0.114–0.134]**	**0.291**	**[0.273–0.309]**

**Fig. 4:**
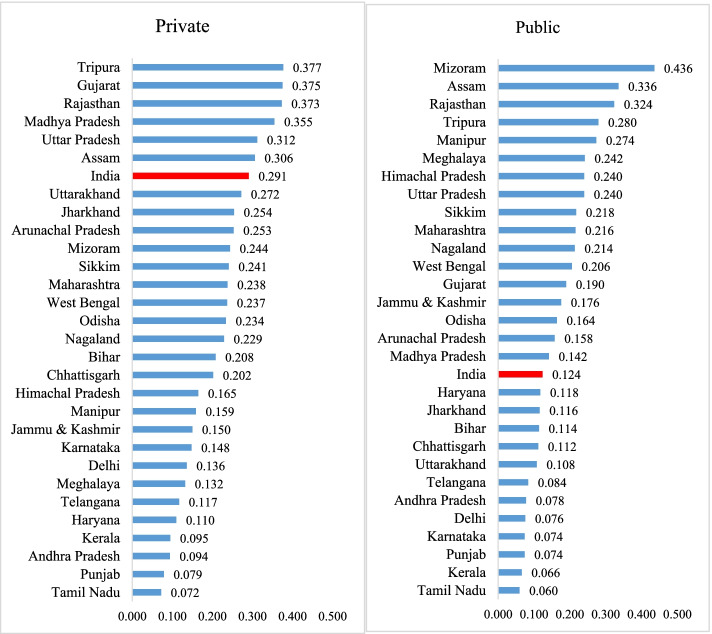
Concentration index of cesarean delivery by type of health facility in selected states of India, 2015–16

**Figure ****4** presents the concentration indices (CI) for cesarean delivery for selected states by type of health facility in India. The inequality in CS delivery was pro-rich in nature in both public and private health facilities in India. Wide variation in CI was observed across the states in both public and private health facilities. With respect to public health facilities, the CI value was highest in Mizoram (0.436) followed by Assam (0.336), Rajasthan (0.324) and Tripura (0.280) while it was lowest in Tamil Nadu (0.060) followed by Kerala (0.066), Punjab (0.074) and Karnataka (0.074). In case of private health facilities, the CI value was the highest in Uttar Pradesh (0.412) followed by Tripura (0.377), Gujarat (0.375) and Rajasthan (0.373) and the lowest in Tamil Nadu (0.072), followed by Punjab (0.079), Andhra Pradesh (0.094) and Kerala (0.095).

## Discussion

Increasing institutional deliveries and providing financial protection to households continue to be twin objectives of public investment under the National Health Mission. Public spending on maternal care in India accounts for over half of the national health budget [45] and is primarily targeted to benefit the poor mothers. Schemes under NHM, namely Janani Suraksha Yojana (JSY), which is a conditional cash transfer scheme, and Janani Shishu Suraksha Karyakaram (JSSK), both of which have been operational for more than a decade, have been successful in creating the demand for and increasing use of institutional delivery. Along with an increased incidence of institutional delivery, there has been a six-fold increase in the share of cesarean birth [27, 46, 47]. Though these schemes offer cash assistance for institutional birth including for cesarean section, the extent of inequity in subsidy distribution across socio-economic groups is not known. To our knowledge, this is the first ever study that has estimated the distribution of public subsidy among mother using cesarean delivery facilities in public health centres in India. The salient finding of the paper are as follows:

First, our findings suggests an underutilization of public health facilities for CS delivery among the poor and an overutilization among the rich., Only 5% deliveries among the poorest mothers, compared to 24% among the richest women, were CS deliveries suggesting the sub-optimal use of public health facilities among the poor. Second, the use of public subsidy on cesarean delivery was pro-rich as confirmed from the benefit incidence analysis. The pattern of utilization and distribution of public subsidy was similar by the level of health facility ( primary and secondary health facilities) with varying magnitudes. These results are supported by the inequality analyses made using concentration curves and concentration indices. Third, the state variations in the inequality of CS are large. The extent of inequality in the use of CS delivery from public health centers was the highest in the state of Mizoram (0.436), followed by Assam (0.336) and the lowest in Tamil Nadu (0.060), followed by Kerala (0.066).

We provide some plausible explanations for our findings. Our key finding regarding the pro-rich utilisation and distribution of subsidy for CS delivery care is consistent with the existing literature [48–51]. This trend can be explained using “Inverse Equity Hypothesis” whereby new medical interventions such as CS delivery are more likely to be adopted by affluent mothers than their poorer counterparts, giving rise to increased health inequalities in their utilization [52]. This may also be due to the lower awareness regarding CS services in public health centers among the poorest and poorer mothers. Another reason may be associated with the ability to pay for CS delivery services which varies largely by the wealth group and plays a vital role in determining the uptake of type of health facility. The indirect cost associated with CS delivery, comprising transportation cost, cost for the travelling person, cost of hospital stay, and miscellaneous fees may restrict mothers from economically weaker section of population from the availing CS delivery at public health facilities.

The pro-rich nature of subsidy distribution for CS delivery can be attributed to the higher educational level of mothers. Mother with higher education attainment are more aware about the facilities and subsidy benefits associated with delivery care compared to their less educated counterparts. Schemes like JSY, JSSK, and other state-specific schemes were launched by the government with the prime motive of benefitting the poor and disadvantaged women delivering in an institution by providing them with cash incentives, The inability of these schemes to adequately identify the actual beneficiaries impact the subsidy distribution [53–55]. Another reason for the inequalities in subsidy distribution may be that mothers from the poorer section of the population are less likely to enrolled under health insurance and reimbursement schemes, which might be another reason for higher inequalities in subsidies have found a pro-poor distribution of public health subsidies on institutional delivery in India, our findings on cesarean delivery reveal that the distribution is pro-rich [30]. This is likely because the costs associated with a CS delivery are higher than those associated with a normal delivery. Some small- scale and unrepresentative studies have found a higher use of CS delivery services among the poorest and poorer wealth quintiles using descriptive analysis.

Despite providing a comprehensive understanding of the distributional aspect of public health subsidies on CS delivery services, our study has some limitations. First, the NFHS data on OOP payment for cesarean delivery may subjected to recall bias. Second, the NFHS data does not provide any information on providers cost of delivery care in a health facility. There is no other source from where we can get such information at the state level where the cost differ significantly. Hence, we had to take the median OOP payment in private health facilities as a proxy of the actual cost of CS delivery in public health facility. Third, it is possible that the utilization and need for CS delivery may varies significantly among mothers from different wealth quintiles, which impacts the subsidy distribution. However, we were unable to perform the need analysis for CS delivery due to a lack of evident information in the data.

## Conclusion

As a signatory to the SDGs, India has made substantial progress in improving its maternal and child health indicators. However, these outcomes are still way off the mark among the poor and marginalized sections of the population. Despite several programs providing financial assistance to poor and marginalized mothers for CS birth, the pro-rich utilization and distribution of public subsidy for CS delivery underlines the inequality in the access to and the outreach of health services, and the inadequate distribution of public health subsidy. Hence, from policy perspective, periodic monitoring and evaluations of the cash incentive schemes for CS delivery is recommended to achieve a more equitable allocation of public health subsidy. Second, increasing the awareness on the availability of cesarean delivery services in public health facilities among the poor and marginalized groups can increase the use of these services. Third, integrating CS delivery services in the newly implemented Ayushman Bharat health insurance schemes for poor mothers can save them from financial catastrophe if they need cesarean delivery.

## Supplementary Information

Additional file 1. Utilization rate, out of pocket payment (₹), and Benefit incidence by mother’s age, and no. of ANC visit on cesarean delivery in India, 2015-16

## Data Availability

The dataset used and analyzed for the current study is open to access publically, require no prior permission, and available in DHS repository [https://dhsprogram.com/data/dataset/India_Standard-DHS_2015.cfm?flag=0].

## Abbreviations

CS: Cesarean Section
WHO: World Health Organization
BMI: Body Mass Index
SDG: Sustainable Development Goals
OOP: Out-of-Pocket Payment
CHS: Catastrophic Health Spending
LMICs: Low and middle-income countries
NHM: National Health Mission
JSY: Janani Suraksha Yojana
JSSK: Janani Shishu Suraksha Karyakaram
PMMVY: Pradhan Mantri Matru Vandana Yojana
NFHS: National Family Health Survey
CEB: Census Enumeration Blocks
PHC: Primary Health Center
UHCs: Urban Health Center
UFWC: Urban Family Welfare Center
UPHC: Urban Primary Welfare Center
BIA: Benefit Incidence Analysis
CC: Concentration Curve
CI: Concentration Index
LPS: Low Performing States
HPS: High Performing States
SC: Scheduled Caste
ST: Scheduled Tribe
OBC: Other Backward Class
CPI: Consumer Price Index
PSU: Primary Sampling Unit
