# Supplementary material for: Are cesarean deliveries equitable in India: assessment using benefit incidence analysis
Source: BMC Health Serv Res. 2022 May 18;22:670. doi: 10.1186/s12913-022-07984-6 (PMC9118745; doi:10.1186/s12913-022-07984-6)
Supplement: Supplementary file 1 — Utilization rate, out of pocket payment (₹), and Benefit incidence by mother’s age, and no. of ANC visit on cesarean delivery in India, 2015-16 [file 12913_2022_7984_MOESM1_ESM.docx]

**Appendix 1:** Utilization rate, out of pocket payment (₹), and Benefit incidence by mother’s age, and no. of ANC visit on cesarean delivery in India, 2015-16.

|  | **Wealth Quintile** | **Number of people utilizing public health services (1)** | **Utilization Rate (2)** | **Median OOP in Public Health services in ₹ (3)** | **Median cost of service in private health center in ₹ (4)** | **Net subsidy at public health center in ₹ (5=4-3)** | **Individual Subsidy Benefit (6=5*2)** | **Benefit Incidence (7)** |
| --- | --- | --- | --- | --- | --- | --- | --- | --- |
| **Mother’s Age (15-24)** | **Poorest** | 456 | 0.115 | 3800 | 20000 | 16200 | 1856 | 10.9 |
|  | **Poorer** | 694 | 0.174 | 3500 | 20000 | 16500 | 2876 | 16.9 |
|  | **Middle** | 891 | 0.224 | 3200 | 20000 | 16800 | 3760 | 22.1 |
|  | **Richer** | 1040 | 0.261 | 3000 | 20000 | 17000 | 4441 | 26.1 |
|  | **Richest** | 900 | 0.226 | 2000 | 20000 | 18000 | 4069 | 23.9 |
|  | **Total** | 3981 |  |  |  |  | 17003 |  |
| **Mother’s Age (25-34)** | **Poorest** | 678 | 0.092 | 4000 | 20000 | 16000 | 1469 | 8.8 |
|  | **Poorer** | 1297 | 0.176 | 4400 | 20000 | 15600 | 2740 | 16.5 |
|  | **Middle** | 1879 | 0.254 | 3000 | 20000 | 17000 | 4326 | 26.0 |
|  | **Richer** | 1987 | 0.269 | 3050 | 20000 | 16950 | 4561 | 27.4 |
|  | **Richest** | 1543 | 0.209 | 3000 | 20000 | 17000 | 3552 | 21.3 |
|  | **Total** | 7384 |  |  |  |  | 16649 |  |
| **Mother’s Age (35 and above)** | **Poorest** | 76 | 0.059 | 2300 | 22200 | 19900 | 1181 | 6.3 |
|  | **Poorer** | 166 | 0.130 | 5000 | 22200 | 17200 | 2229 | 11.8 |
|  | **Middle** | 314 | 0.245 | 3300 | 22200 | 18900 | 4633 | 24.5 |
|  | **Richer** | 413 | 0.322 | 3500 | 22200 | 18700 | 6029 | 31.9 |
|  | **Richest** | 312 | 0.244 | 2400 | 22200 | 19800 | 4822 | 25.5 |
|  | **Total** | 1281 |  |  |  |  | 18894 |  |
| **ANC visit (Less than 4 visit)** | **Poorest** | 354 | 0.094 | 3200 | 19820 | 16620 | 1561 | 9.3 |
|  | **Poorer** | 504 | 0.134 | 4900 | 19820 | 14920 | 1995 | 11.9 |
|  | **Middle** | 748 | 0.198 | 4500 | 19820 | 15320 | 3040 | 18.2 |
|  | **Richer** | 1007 | 0.267 | 2200 | 19820 | 17620 | 4708 | 28.1 |
|  | **Richest** | 1156 | 0.307 | 2100 | 19820 | 17720 | 5435 | 32.5 |
|  | **Total** | 3769 |  |  |  |  | 16739 |  |
| **ANC visit (4 & more )** | **Poorest** | 1224 | 0.138 | 4000 | 20100 | 16100 | 2220 | 13.1 |
|  | **Poorer** | 1905 | 0.215 | 3300 | 20100 | 16800 | 3605 | 21.3 |
|  | **Middle** | 2223 | 0.250 | 3000 | 20100 | 17100 | 4282 | 25.3 |
|  | **Richer** | 2018 | 0.227 | 3100 | 20100 | 17000 | 3865 | 22.8 |
|  | **Richest** | 1507 | 0.170 | 2700 | 20100 | 17400 | 2954 | 17.5 |
|  | **Total** | 8877 |  |  |  |  | 16926 |  |
| **No Complication** | **Poorest** | 450 | 0.079 | 3600 | 20000 | 16400 | 1297 | 7.69 |
|  | **Poorer** | 883 | 0.155 | 4000 | 20000 | 16000 | 2483 | 14.72 |
|  | **Middle** | 1421 | 0.250 | 3200 | 20000 | 16800 | 4195 | 24.87 |
|  | **Richer** | 1633 | 0.287 | 3300 | 20000 | 16700 | 4792 | 28.41 |
|  | **Richest** | 1304 | 0.229 | 2100 | 20000 | 17900 | 4101 | 24.32 |
|  | **Total** | 5691 |  |  |  |  | 16868 |  |
| **Any Complication** | **Poorest** | 746 | 0.107 | 4000 | 20000 | 16000 | 1716 | 10.24 |
|  | **Poorer** | 1232 | 0.177 | 3500 | 20000 | 16500 | 2923 | 17.44 |
|  | **Middle** | 1662 | 0.239 | 3000 | 20000 | 17000 | 4062 | 24.24 |
|  | **Richer** | 1808 | 0.260 | 3000 | 20000 | 17000 | 4419 | 26.37 |
|  | **Richest** | 1507 | 0.217 | 3200 | 20000 | 16800 | 3640 | 21.72 |
|  | **Total** | 6955 |  |  |  |  | 16761 |  |
